# Interactions between parasitic helminths and gut microbiota in wild tropical primates from intact and fragmented habitats

**DOI:** 10.1038/s41598-021-01145-1

**Published:** 2021-11-03

**Authors:** Claudia Barelli, Claudio Donati, Davide Albanese, Barbora Pafčo, David Modrý, Francesco Rovero, Heidi C. Hauffe

**Affiliations:** 1 Conservation Genetic Research Unit, Research and Innovation Centre, Fondazione Edmund Mach, S. Michele All’Adige, Italy; 2 Department of Biology, University of Florence, Sesto Fiorentino, Italy; 3 Computational Biology Research Unit, Research and Innovation Centre, Fondazione Edmund Mach, S. Michele All’Adige, Italy; 4 Department of Pathology and Parasitology, University of Veterinary Sciences, Brno, Czech Republic; 5 Institute of Vertebrate Biology, Czech Academy of Sciences, Brno, Czech Republic; 6 Biology Centre, Institute of Parasitology, Czech Academy of Sciences, Ceske Budejovice, Czech Republic; 7 Department of Botany and Zoology, Faculty of Science, Masaryk University, Brno, Czech Republic

**Keywords:** Biodiversity, Bacteria, Fungi, Parasitology

## Abstract

The mammalian gastrointestinal tract harbours a highly complex ecosystem composed of a variety of micro- (bacteria, fungi, viruses, protozoans) and macro-organisms (helminths). Although most microbiota research focuses on the variation of single gut components, the crosstalk between components is still poorly characterized, especially in hosts living under natural conditions. We investigated the gut micro-biodiversity (bacteria, fungi and helminths) of 158 individuals of two wild non-human primates, the Udzungwa red colobus (*Procolobus gordonorum*) and the yellow baboon (*Papio cynocephalus*). These species have contrasting diets and lifestyles, but live sympatrically in both human-impacted and pristine forests in the Udzungwa Mountains of Tanzania. Using non-invasive faecal pellets, helminths were identified using standard microscopy while bacteria and fungi were characterized by sequencing the V1–V3 variable region of the 16S rRNA gene for bacteria and the ITS1–ITS2 fragment for fungi. Our results show that both diversity and composition of bacteria and fungi are associated with variation in helminth presence. Although interactions differed by habitat type, in both primates we found that *Strongyloides* was negatively associated and *Trichuris* was positively associated with bacterial and fungal richness. To our knowledge, this is one of the few studies demonstrating an interaction between helminth and gut microbiota communities in wild non-human primates.

## Introduction

Healthy mammalian gastrointestinal tracts harbour a complex ecosystem where micro- (i.e. bacteria and fungi) and macrobiota (i.e. protists and helminths) co-exist and have co-evolved in association with their mutual hosts^1,2^. As the bacterial community exceeds all other components within the gut in terms of number and species diversity^3^, our knowledge of the microbiota is shaped by a disproportionate focus on this gut component. A plethora of evidence suggests that a rich and diverse gut bacterial community is beneficial to host health^4–6^ by maintaining important metabolic and immune functions^7–9^, and contributing to colonization resistance against enteric pathogens^10–12^. However, other relevant (but mostly neglected) gut components are also potentially beneficial or neutral depending on species and context. For example, although they have been mainly studied for their pathogenic characteristics^13^, gut helminths secrete an array of molecules that can directly affect host immune response^14^, or may do so indirectly by interacting with the gut microbiota by modifying bacterial richness and diversity^15–19^.

Although the field is developing quickly, little information is available on the details of micro-/macrobiota interactions in the gut, and interactions of this microdiversity with the host^20^. The majority of studies focussing on this topic derive from laboratory animal experiments^21–23^, but few observations in humans^24,25^ and wild non-human animals^19,26^ have revealed associations between gut bacterial communities and helminth infection (but see^27,28^). Due to the potential beneficial consequences of such interactions for the overall health and well-being of the hosts (e.g. dampening allergic responses and ameliorating of gut dysfunction in humans^29^), investigating the gut communities of animal species closely related to humans (i.e. non-human primates) living under natural conditions is of particular relevance. Studies of these associations will provide a critical foundation to understanding the potential mechanisms that underpin the interplay between helminths, microbiota and hosts, and will deepen our awareness of animal health with possible conservation implications.

Non-human primates represent a crucial element of tropical ecosystems, contributing to forest regeneration and ecosystem health^30^. However, they are facing escalating loss and fragmentation of optimal habitat, making this mammalian group one of the most threatened on Earth^31^. Recent research we conducted on primates living sympatrically in human-modified and pristine habitats has highlighted the impact of habitat changes on gut bacterial and fungal diversity^32,33^, as well as on helminth infections^34,35^. Although a few recent studies have evaluated the diversity and composition of the gut bacterial communities of non-human primates naturally infected by helminths^36–39^, here we compare both the bacterial and fungal components in helminth-positive and helminth-negative individuals to investigate: (i) whether bacterial and fungal richness and/or composition vary in primates with different ecological adaptations residing in either pristine or human-impacted forests; (ii) which bacterial and fungal taxa are responsible for such variation; and (iii) which of the detected helminths interplay with bacterial and fungal richness.

## Results

This paper investigated the interactions between helminth presence and gut microbiota components (bacteria and fungi) in two primate species (red colobus monkeys and yellow baboons) living in two forest types (intact and fragmented). As reported in^35^, of 158 faecal samples collected from 89 red colobus and 69 yellow baboons 76.6% (63.3% for red colobus and 95.6% for yellow baboons, respectively) were positive for eggs of at least one of the five helminth types identified, two of which (nematodes) could be classified to genus (*Strongyloides*, *Trichuris*), while another three to higher taxonomic levels (dicrocoeliid trematodes, and spirurid and strongylid nematodes). No metazoan larvae or adults were detected with the gauze-washing method. Instead^32^, showed that the most prevalent bacterial phyla in both red colobus and baboons were Firmicutes, Bacteroidetes, Proteobacteria and Spirochetes, accounting for 75.3% and 84.6% of the total bacterial amplicons, respectively; the four prevalent and classified phyla of fungi were Ascomycota, Basidiomycota and Zygomycota for both host species.

**Figure 1:**
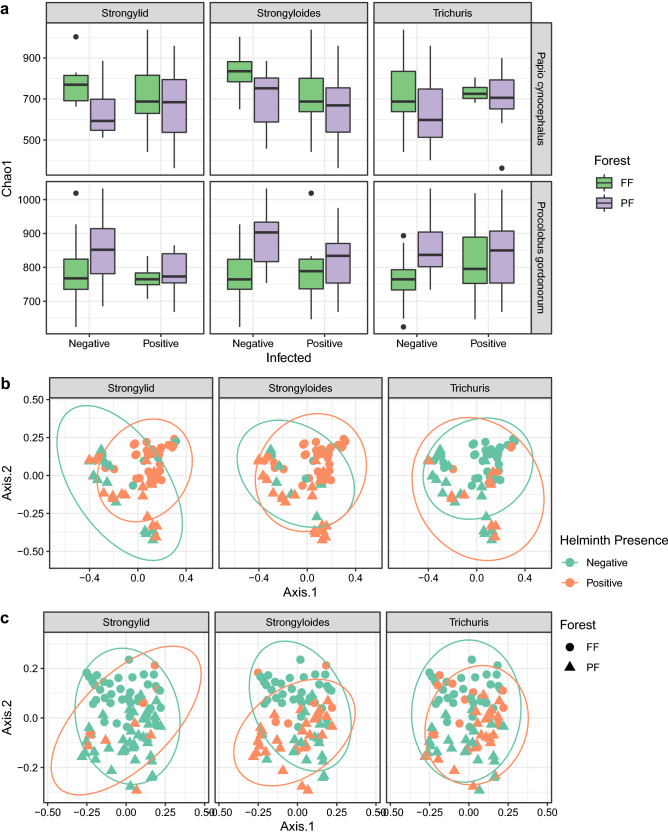
Diversity of gut bacterial communities in yellow baboons and Udzungwa red colobus in two forest types. (**a**) Comparison of bacterial richness measured by Chao1 estimator and (**b**,**c**) bacterial composition measured by Bray–Curtis dissimilarities for helminth negative and positive faecal samples. Panels from left to right focus on three helminth taxa: strongylid nematodes, *Strongyloides*, and *Trichuris* present in yellow baboons, *Papio cynocephalus* [upper panel in (**a**); (**b**)], and Udzungwa red colobus monkeys, *Procolobus gordonorum* [bottom panel in (**a**); (**c**)], living in the forest fragment of Magombera [FF: green in (**a**), circle in (**b**) and (**c**)] or the protected forest of Mwanihana [PF: purple in (**a**); triangle in (**b**) and (**c**)] in the Udzungwa Mountains of Tanzania. In (**a**), the horizontal line in the boxplot indicates the median, the box extends to the 25th–75th percentile and the whiskers extends to the largest value no further than 1.5 * IQR from the hinges. In (**b**) and (**c**) colours represent helminth negative (green) and positive (orange) samples.

Our analysis here showed that helminth presence was associated with gut microbiota richness and composition for three of the five helminth taxa found in the two primate species studied here: *Strongyloides*, strongylid nematodes, and *Trichuris*. The same three taxa were also the most prevalent helminth types, both in yellow baboons (85.5%, 75.4%and 23.4%, respectively) and Udzungwa red colobus (43.9%, 11.2% and 38.8%, respectively; see details in^35^). Specifically, yellow baboons helminth-positive for strongylid nematodes had lower bacterial richness than helminth-negative individuals living in the Magombera fragmented forest (hereafter FF: fragmented forest) (P = 2.03 × 10^–7^, helminth-positive: N = 6, helminth-negative: N = 33, Tukey post-hoc test, see “Methods” section; Fig. 1a), but the opposite association was found in the Mwanihana protected forest (PF) (i.e. individuals helminth-positive for strongylid nematodes had higher bacterial richness; P = 0.01, helminth-positive: N = 19, helminth-negative: N = 11). Instead, baboons helminth-positive for *Strongyloides* had lower bacterial richness than helminth-negative individuals in both FF (P = 5.30 × 10^–15^, helminth-positive: N = 35, helminth-negative: N = 4; Fig. 1a) and PF (P = 9 × 10^–6^, helminth-positive: N = 24, helminth-negative: N = 6; Fig. 1a). In addition, a positive association between *Trichuris* and bacterial richness was also found in individuals living in PF (P = 1.26 × 10^–12^, helminth-positive: N = 12, helminth-negative: N = 18; Fig. 1a).

Similar to the yellow baboon, for red colobus monkeys, we detected associations between bacterial richness and helminth presence for the same three helminth taxa: strongylid nematodes, *Strongyloides* and *Trichuris*. Specifically, we found negative associations between the presence of both strongylid nematodes and *Strongyloides* and bacterial richness in animals from PF (P = 9.84 × 10^–9^, helminth-positive: N = 4, helminth-negative: N = 40; and P = 1.69 × 10^–10^, helminth-positive: N = 7, helminth-negative: N = 37, respectively; Fig. 1a), while the presence of *Trichuris* was positively associated with bacterial richness in red colobus living in FF (P = 1.44 × 10^–7^, helminth-positive: N = 12, helminth-negative: N = 32; Fig. 1a; see Table S1 for details). Using Shannon entropy as a measure of bacterial diversity, we found no significant differences associated with helminth presence (Table S2).

Analyses of the interactions between gut microbiota composition, measured by Bray–Curtis dissimilarities, and helminth presence/absence were less clear cut (Fig. 1b,c): using PERMANOVA we found that only *Strongyloides* in red colobus monkeys appeared to be associated with gut bacterial composition between individuals infected and non-infected with these nematodes in FF (P = 0.003; Fig. 1c). DESeq2 results showed that among the bacterial taxa that could explain this difference, 17 were enriched in the animals infected with *Strongyloides*; 11 of these belonged to the order Clostridiales (mainly Lachnospiraceae and Ruminococcaceae families), two to the Bacteroidales and the remaining four were unclassified (Table S3). Among the genera identified, *Pseudoflavonifractor* was overrepresented in the red colobus infected by *Strongyloides*.

**Figure 2:**
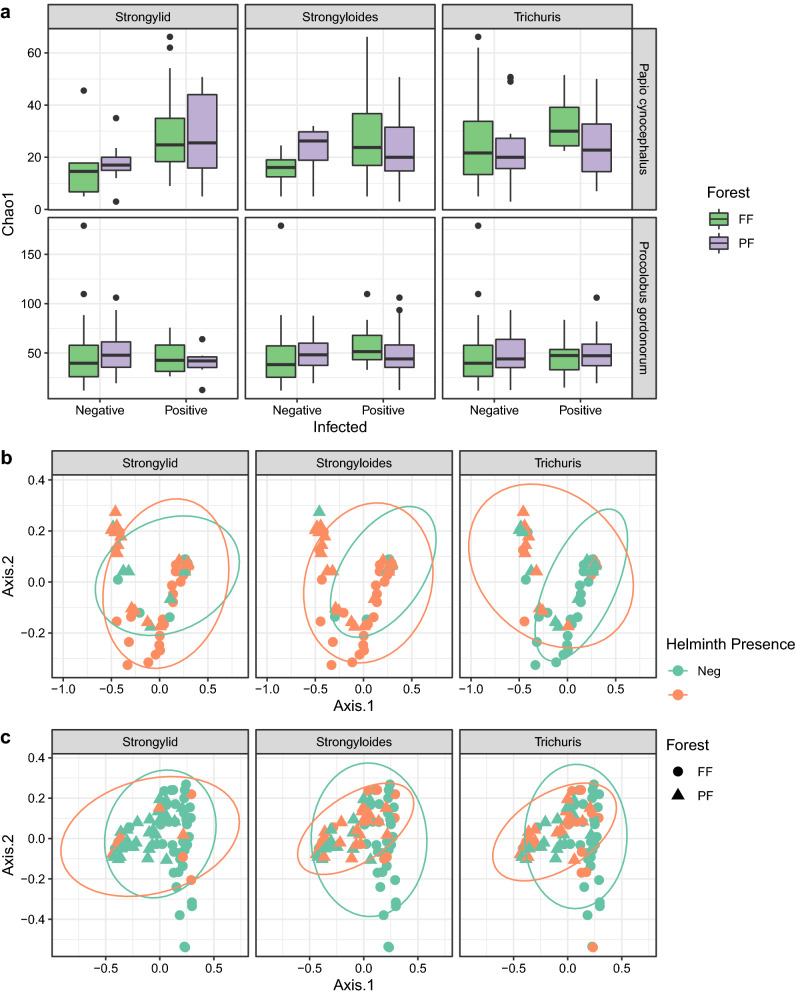
Diversity of gut fungal communities in yellow baboons and Udzungwa red colobus in different forest types. (**a**) Comparison of fungal richness measured by Chao1 estimator and (**b**,**c**) fungal composition measured by Bray–Curtis dissimilarities for helminth negative and positive faecal samples. Panels from left to right focus on three helminth taxa: strongylid nematodes, *Strongyloides*, and *Trichuris* present in yellow. In (**a**), the horizontal line in the boxplot indicates the median, the box extends to the 25th–75th percentile and the whiskers extends to the largest value no further than 1.5 * IQR from the hinges. In (**b**) and (**c**) colours represent helminth negative (green) and positive (orange) samples.

As for bacteria, using a GLM (see “Methods”) to model the effect of helminth presence on fungal richness, we found a positive association between fungal richness and the presence of three helminth types (strongylid nematodes: P = 6.92 × 10^–7^, post-hoc Tukey test, helminth-positive: N = 33, helminth-negative: N = 6; *Strongyloides*: P = 4.35 × 10^–6^; *Trichuris*: P = 0.006, helminth-positive: N = 35, helminth-negative: N = 4; Fig. 2a, upper panel) in yellow baboons living in FF. Moreover, we found a positive association between fungal richness and the presence of strongylid nematodes in baboons from PF (P = 6.89 × 10^–7^, helminth-positive: N = 19, helminth-negative: N = 11; Fig. 2a, upper panel). For red colobus monkeys, we found a negative association between fungal richness and strongylid presence in animals living in PF (P = 1.36 × 10^–4^, helminth-positive: N = 4, helminth-negative: N = 40; Fig. 2a, lower panel) as well as a positive association between fungal richness and *Strongyloides* presence in animals from FF (P = 2.73 × 10^–7^, helminth-positive: N = 7, helminth-negative: N = 37; Fig. 2a, lower panel). All other comparisons were not statistically significant (Table S5). As for bacteria, we found no significant association between helminth presence and fungal diversity measured by Shannon entropy (Table S5).

Similarly to bacteria, limited associations were observed between the fungal composition and helminth positive and negative individuals. For both red colobus monkeys and yellow baboons individuals positive and negative for *Trichuris* showed different gut fungal compositions (P = 0.015 and 0.03 respectively, Fig. 2b,c). However, in both cases a significant inhomogeneity of dispersions around centroids was identified (ANOVA test P = 0.017 and 0.010, respectively). DESeq2 identified the family Xylariaceae to be enriched in individuals infected with *Trichuris* (Table S6).

## Discussion

Here we present the associations between gut bacterial and fungal communities (using 16S rRNA and ITS1-ITS2 amplicon sequencing) and the presence or absence of helminths (measured as shedding of eggs via standard flotation methods and optical microscopy) in two species of wild non-human primates living in the same intact and human-modified tropical forest blocks. We found that changes in gut microbiota richness and diversity in both primate species were associated with the presence of certain helminth types, but that this variation was also affected by habitat. Given the known effects of low gut microbiota diversity in human health and laboratory animals, the loss of natural levels of micro-biodiversity could have wide-ranging and devastating effects on the survival of wild animal species, but the drivers and rate of loss in free-living populations is virtually unknown. Our study provides a novel addition to the rare studies^36,37,39^ investigating the associations between bacterial communities and helminth presence in wild non-human primates, and, for the first time, including fungal communities in the analyses.

To the best of our knowledge this is the first study that uses ITS1-2 Illumina MiSeq technology to characterize gut fungal communities and associate them with helminth presence.

To better understand the biological meaning of the observed associations, we restricted our discussion here to the two soil-transmitted helminths, namely *Trichuris* sp. and *Strongyloides* sp. since these taxa are present in most social groups of both primate species (94.6% and 77.2% of individuals, including all social groups for yellow baboon and red colobus, respectively), and more thoroughly discussed in the literature than the broader taxonomic helminth groups that could be identified here. Helminths of both genera parasitize a wide variety of different mammalian hosts^40,41^, although they have different life cycles. Embryonated eggs of *Trichuris* spp. are shed in the faeces; larvae develop inside the eggs in a moist environment and are ingested by new hosts with food, water, and soil. Once in the small intestine, *Trichuris* eggs are stimulated by the cecal microbiota to hatch and first-stage larvae embed themselves into the mucosa of the large intestine; after mating, female whipworms produce eggs to complete the cycle^42^. Instead, *Strongyloides* leave the host either as first stage larvae or embryonated eggs shed into the environment, but go through several free-living stages before being ingested, and inhabiting the mucosa of the small intestine^41,43^. Previous studies of bacterial microbiota and the soil-transmitted *Trichuris* and *Strongyloides* helminths have reported various results, from no association (i.e. *Trichuris* sp. in rural Ecuadorian children^28^; *Strongyloides* sp. in wild western chimpanzee^44^) to alteration of the gut microbiota, by reducing^16,45^ or increasing^24,46,47^ bacterial richness and diversity. We found robust patterns of associations between bacterial and fungal components of the microbiota and helminths across the two primate species. Bacterial and fungal richness increased, and fungal composition changed when individual primates were infected by *Trichuris*, while bacterial richness declined in individuals infected with *Strongyloides*. This was generally true for both the leaf-eating, arboreal red colobus monkey and the omnivorous, terrestrial yellow baboon, although habitat also had some effect. Presence of strongylid nematodes, *Strongyloides* or *Trichuris* all increased fungal richness in yellow baboons in FF, while nematode presence (strongylid nematodes, *Strongyloides*) was associated with lower bacterial richness in this species in the same habitat. Nematode presence (strongylid nematodes, *Strongyloides*) was also associated with lower bacterial richness in red colobus, but in PF. Although studies of the interactions between helminths and microbiota in wild animal populations are still relatively new, we speculate that the discrepancy found here may derive from a temporary instability in microbiota composition as a result of helminth infections which may recover over time, as already found in humans under controlled settings^17^. Further longitudinal studies on the same wild populations could help to fulfil this gap and reveal the actual influence of each gut component over time.

However, it has been suggested previously that a high level of bacterial richness and diversity is considered a desirable trait^5,48^. Moreover, *Trichuris* infection is known to ameliorate inflammatory bowel disorders in humans^49^. As shown in studies conducted on humans^24,46^ and in a pioneer study on non-human primates^18^, *Trichuris* infections were also beneficial to restoring bacterial diversity. Indeed, after being therapeutically infected with *Trichuris* gut bacterial communities were reversed in juvenile rhesus macaques (*Macaca mulatta*) with colitis as bacterial richness was restored to levels comparable to those of healthy macaques^18^. Interestingly, our findings in wild primates agree with these results, showing a positive association between *Trichuris* presence and bacterial richness. The positive interaction between *Trichuris* and bacterial richness we found in Udzungwa red colobus inhabiting FF are particularly intriguing, since these same populations have been shown to suffer loss of bacterial diversity due to the poorer diet (i.e. lower tree diversity compared to intact forests) in this degraded habitat^32,33^. Similarly, the same association found in yellow baboons was restricted to individuals living in the pristine forest, that have a lower bacterial richness compared to animals living in the human-modified forest, where they supplement their diet by raiding crops and feeding on human organic waste. Thus, positive associations between *Trichuris* presence and bacterial richness in our wild primate models may reflect an ecological strategy to ameliorate low gut microbiota.

In contrast to the results for *Trichuris*, both Udzungwa red colobus (in PF) and yellow baboons (in both PF and FF) positive for *Strongyloides* had lower bacterial richness compared to *Strongyloides* negative ones. This apparently contradicts the studies conducted in human and non-human primates which found a positive association^46,47^ or no association^44^ between *Strongyloides* and bacterial richness. However, an experimental study conducted on mice infected with *S. venezuelensis* revealed a reduction in bacterial richness at peak of infection^45^, reflecting temporary bacterial alteration potentially due to gut immune responses. Therefore, we cannot exclude the possibility that the lower bacterial richness in *Strongyloides-*individuals observed here also reflect a transitory effect characteristic of this helminth in any habitat.

*Strongyloides* secrete a variety of proteins in the host gut, which have antimicrobial activities leading to alteration in bacterial diversity and composition as also recently observed in laboratory mice^50^. In fact, while we observed reduction of gut bacterial diversity in association with *Strongyloides* infection, the concurrent change in beta diversity (reflecting a variation in bacterial composition) shows that primate species respond differently to helminth presence. Changes in bacterial composition for the Udzungwa red colobus in PF appears to have been driven by Lachnospiraceae and Ruminococcaceae bacterial families. Both families are associated with degradation of complex plant material like cellulose and lignin^51,52^, which may well reflect their folivorous diet, as also found in most of the leaf-eating primates and ruminants^53,54^. However, although both families are associated with energy metabolic advantages, their high abundance has been related to metabolic disorders^55,56^. Moreover, the genus *Pseudoflavonifractor,* a biomarker of obesity and diabetes, was also overrepresented in individuals infected by *Strongyloides*, implying that, besides being associated with an overall reduction of bacterial richness, *Strongyloides* may contribute to the emergence of less beneficial bacterial taxa and thus, to the disruption of the metabolic balance within a healthy gut^57^.

No studies in either experimental and natural settings are available on the interaction between gut fungal communities and the helminths taxa we identified here, although some human models have shown that fungal taxa may be positively associated with parasitic colonization by *Blastocystis*^58^, such as *Saccharomyces boulardii*^59^, as well as *Candida albicans* and *C. glabrata*^60^. Thus, our findings are pioneering this field. We found, for example, that similarly to bacterial microbiota, both *Trichuris* and *Strongyloides* helminths were positively associated with fungal richness in both yellow baboons and Udzungwa red colobus living in FF. However, since no data are available for comparison with our results, and the mechanism behind these associations is difficult to disentangle, we can only conclude that fungal richness, similarly to that of bacteria, is probably highly relevant for gut function^61^. However, the notion that either a competitive or a beneficial association between gut components exists cannot be verified at this time.

## Conclusions

Although causal relationships must be further investigated, the current study underlines the importance of considering the associations between gut components in investigations of wild animal health and conservation status, especially in endangered species threatened by increasing human pressure. Our findings revealed that environmental factors may play an important role in shaping such associations. Thus, to clarify to what extent biological and environmental variables are interconnected, their transmission potential and the direct consequences on animal status, future studies would benefit from better classification of each gut component and determination of their metabolic functions, as well as broadening the investigations to include additional host gut components (e.g. viruses), and the microbiota of their surrounding ecosystem (e.g. sympatric hosts, food web, soil, water sources).

## Methods

### Study site and animal populations

**Figure 3:**
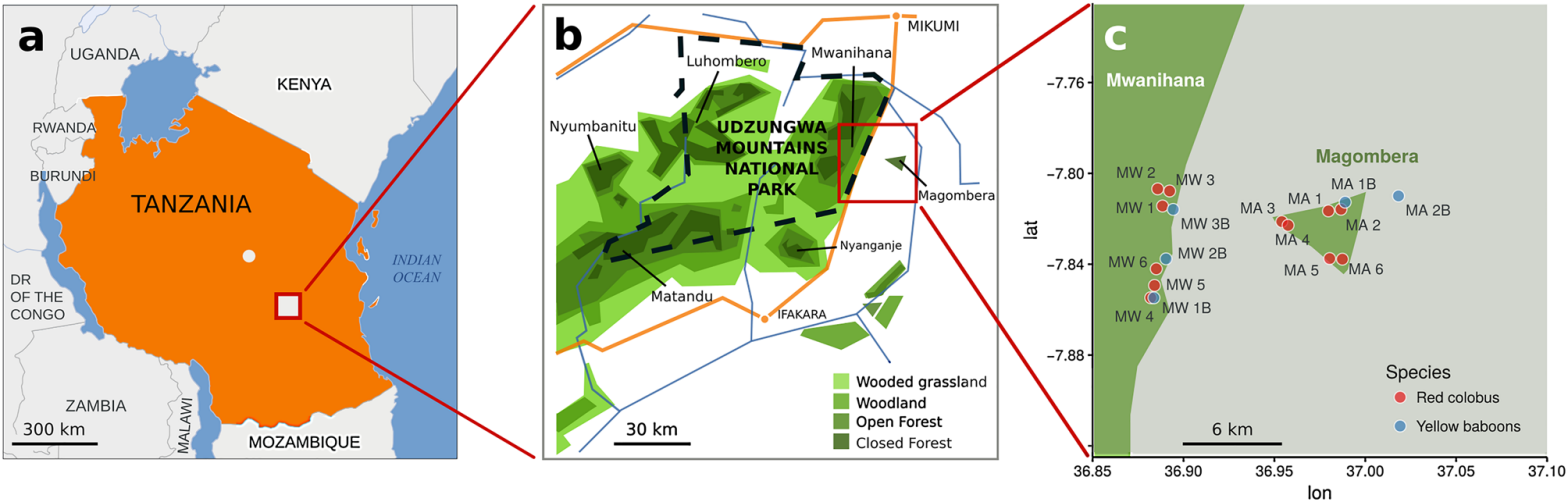
Map of the study site in the Udzungwa Mountains, Tanzania (**a**). Enlargement indicating the forested areas considered in this study: Mwanihana (protected forest) and Magombera (fragmented forest block) (**b**). Sample sites for Udzungwa red colobus (red circles: 12 social groups) and yellow baboons (blue circles: five social groups) (**c**). Dashed line in (**b**) indicates the border of the Udzungwa Mountains National Park. Original map reproduced with permission from^32^.

We sampled faecal pellets of the Udzungwa red colobus (*Procolobus gordonorum*) and yellow baboon (*Papio cynocephalus*) following^32^ in the Udzungwa Mountains of Tanzania (7°40′ S to 8°40′ S and 35°10′ E to 36°50′ E; Fig. 3), an internationally-recognized biodiversity hotspot^62^ extending over 19,000 km^2^^63^. The Udzungwa Mountains are characterized by several disjunct forest blocks with different levels of protection (from national park to poorer protection in other types of reserve), which affect the conservation status of the primate populations living within them^64,65^. Remaining forests are threatened by intensive agriculture^66^, and primates outside the Udzungwa Mountains National Park (established in 1992 with an area of 150 km^2^ and elevational range from 351 to 2263 m a.s.l.) are particularly vulnerable to hunting and other human disturbance^67^.

Our two focal primate species differ in ecological niche and dietary strategy: the Udzungwa red colobus is arboreal and feeds mainly on leaves and stems, while the yellow baboon is terrestrial and omnivorous, foraging for arthropods, seeds, fruit and leaves, opportunistically raiding crops. The red colobus is endemic and IUCN-Vulnerable, while the yellow baboon is categorized as ‘Least Concern’^68^. Both these species live in PF and FF. PF is an intact semi-deciduous to sub-montane and montane evergreen forest that lies in the eastern part of the Udzungwa Mountains National Park, while FF is flat, groundwater forest surrounded by villages and sugarcane plantations, approximately 6 km east of PF (area extension: 12 km^2^, altitude: 269 to 302 m a.s.l.; Figs. S1 and S2).

### Sample collection, parasitological assessment and gut microbiota detection

To assess the diversity of and interactions between the helminth, fungal and bacterial components of the gut flora of the red colobus and yellow baboon, in June and July 2016 we followed social groups unobtrusively and collected freshly deposited faecal samples from the ground for 89 red colobus (belonging to 12 social groups, six in PF and six in FF) and 69 yellow baboons (five social groups, 3 from PF, 2 from FF; Fig. 3c) following^32,33^ methods.

Each faecal sample was divided in two parts: the subsamples used to identify helminths were preserved in 10 ml of 10% neutral buffered formalin and stored at ambient temperature (20–25 °C) until transport to the Fondazione Edmund Mach (FEM), Italy where they were stored at 4 °C before shipment to the University of Veterinary Sciences in Brno, Czech Republic, for parasitological analysis. Helminth identification was carried out using both modified Sheather’s flotation and faecal sedimentation techniques^69,70^. In brief, from the whole sediment sample (see details in^35^) we examined 2 ml of faecal suspension using optical microscopy at 400× magnification (OLYMPUS CX40, Japan). Based on morphological characteristics, eggs were identified for each helminth taxon. Lastly, using what was left of the faecal sample, adult and larval helminths were collected using the ‘gauze-washing’ method^71^ and observed via stereo microscopy (OLYMPUS SZ51, Japan) at 8–40× magnification.

The faecal subsamples used for microbiota analysis were preserved in 96% ethanol and kept at 4 °C before transporting them to FEM, stored at − 80 °C and shipped to the University of Illinois, USA for analysis (described in^32^). Briefly, from each faecal sample, we extracted and amplified the whole DNA using the same protocols^72,73^, and later sequenced the V1-V3 regions of the 16S ribosomal RNA gene for bacteria and the ITS1-ITS2 region for fungi using Illumina MiSeq technology. The software MICCA^74^ was used to identify amplicon sequence variants (SVs).

### Statistical analyses

All statistical analyses were conducted using the *R 4.0.2* statistical package^75^. Alpha and beta diversity indices of the bacterial and fungal components of the microbiota were calculated using the package *phyloseq 1.32.0.* Standard alpha diversity index (Chao1 estimator of species richness and Shannon entropy) for both bacteria and fungi were estimated and used them as dependent variables in regression models testing the relationship between gut microbiota (bacterial and fungal) richness and helminth presence separately for each primate species in each forest type (PF and FF). To identify statistically significant effects of infection by helminths on bacterial and fungal richness we performed a post-hoc analysis on marginal means estimated using a Generalized Linear Model (GLM) that guarantees robust results for unbalanced study groups. Specifically, species richness (Chao1) and diversity (Shannon) of bacteria and fungi were used as dependent variables in a GLM with Poisson error distribution, with helminth presence (‘Positive’) or absence (‘Negative’) for dicrocoeliid trematodes, spirurid and strongylid nematodes, *Strongyloides*, and *Trichuris* as independent variables. The model used was:

$$ (Chao1/Shannon\,\sim \,Species\, + \,Parasite\, + \,Forest\, + \,Infected \, \% in\% \, \left( {Species\, + \,Parasite\, + \,Forest} \right) $$

where *Species* indicates the primate species, *Parasite* indicates the helminth type, and *Forest* indicates PF or FF, and *Chao1/Shannon* indicate either Chao1 or Shannon entropy. Contrasts between marginal means for ‘Positive’ and ‘Negative’ groups for each primate species, parasite and forest and significant test were computed using the package *emmeans 1.5.3* on the fitted model^76^. The effect of helminth presence on beta diversity of bacteria and fungi was assessed by PERMANOVA using the function *adonis2* from the *vegan 2.5-7* package. Homogeneity of dispersions was controlled by ANOVA of the multivariate dispersions of the groups obtained using the function *betadisper* from the *vegan 2.5-7* package. DESeq2 was used to identify which bacterial and fungal taxa explained the differences observed in their overall relative abundance between helminth infected and non-infected individuals.

### Ethics statement

The authors confirm they did not interact with or disrupt any of the primate species surveyed in any way. Faecal sample collection was non-invasive, without direct contact or interaction with the animals. Highly trained fieldworkers strictly adhered to the ‘Code of Best Practices for Field Primatology’ published by the International Primatological Society (IPS) as well as the ‘Principles for the Ethical Treatment of Primates’ of the American Society of Primatologists (ASP). Data collection complied with legal requirements and laws governing wildlife research in Tanzania. Research permits (2016-267-ER-2009-49) were obtained through the Tanzania Commission for Science and Technology (COSTECH), Tanzania Wildlife Research Institute (TAWIRI) and Tanzania National Parks (TANAPA).

## Supplementary Information

Supplementary Information 1.

Supplementary Tables.

## Data Availability

DNA sequences have been deposited in the European Nucleotide Archive (ENA) under study accession number PRJEB37770. 16S and ITS raw sequences generated for this study and metadata are publicly available at https://doi.org/10.5281/zenodo.5482908. Scripts for statistics are available at https://github.com/compmetagen/barelli_et_al_scirep_2021.
