# Supplementary material for: Interactions between parasitic helminths and gut microbiota in wild tropical primates from intact and fragmented habitats
Source: Sci Rep. 2021 Nov 3;11:21569. doi: 10.1038/s41598-021-01145-1 (PMC8566450; doi:10.1038/s41598-021-01145-1)
Supplement: Supplementary file 1 — Supplementary Information 1. [file 41598_2021_1145_MOESM1_ESM.pdf]

Interactions between parasitic helminths and gut microbiota in wild tropical primates from intact and fragmented habitats

Supplementary Information

Claudia Barelli[1,2,*], Claudio Donati[3], Davide Albanese[3], Barbora Pafčo[4,5], David Modrý[4,6,7] Francesco Rovero[2] & Heidi C. Hauffe[1]

* Corresponding Author

[1] Department of Biodiversity and Molecular Ecology, Research and Innovation Centre, Fondazione Edmund Mach, Via E. Mach 1, 38098 S. Michele all'Adige, Italy

[2] Department of Biology, University of Florence, Via Madonna del Piano 6, 50019 Sesto Fiorentino, Italy

[3] Computational Biology Research Unit, Research and Innovation Centre, Fondazione Edmund Mach, Via E. Mach 1, 38098 S. Michele all'Adige, Italy

[4] Department of Pathology and Parasitology, University of Veterinary Sciences, Brno, Czech Republic

[5] Institute of Vertebrate Biology, Czech Academy of Sciences, Brno, Czech Republic

[6] Biology Centre, Institute of Parasitology, Czech Academy of Sciences, České Budějovice, Czech Republic

[7] Department of Botany and Zoology, Faculty of Science, Masaryk University, Brno, Czech Republic

* Correspondence to barelli.cla@gmail.com (CB)

Running Title: Host-helminth-microbiota interactions in tropical primates.

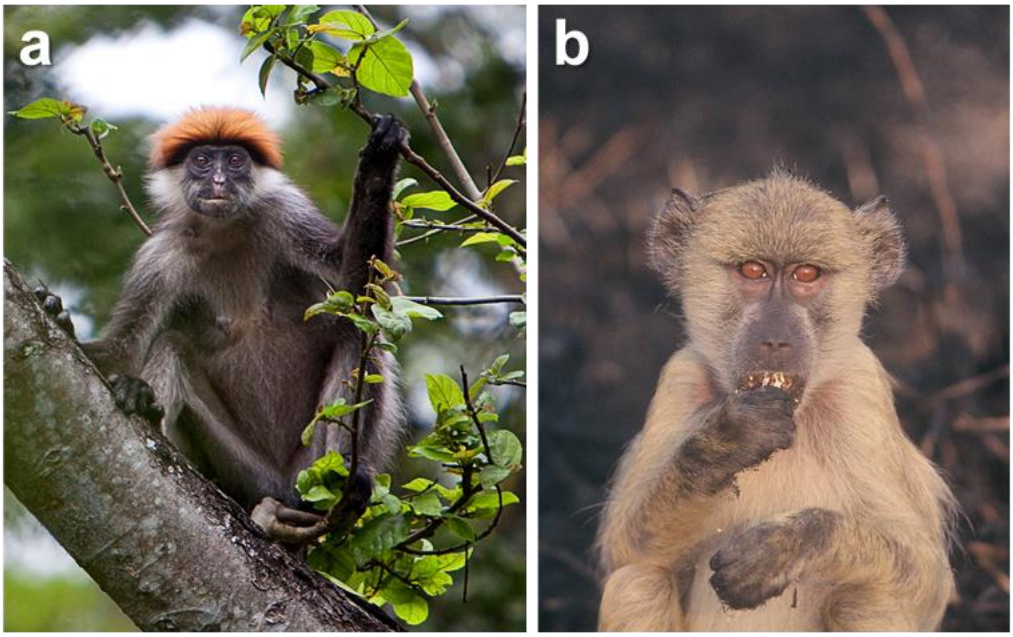

Photo courtesy of Raffaele Merler (**a**) and Francesco Rovero (**b**)

Supplementary Figure S1. Udzungwa red colobus monkeys (*Procolobus gordonorum*) and yellow baboons (*Papio cynocephalus*) living in the Udzungwa Mountains (Tanzania). Udzungwa red colobus are arboreal forest dwellers, endemic and endangered. They have highly specialized digestive system similar to ruminants, they are foregut fermenters and consume preferentially fresh young shoots and leaves. Yellow baboons are mainly terrestrial, highly common and of least conservation concern. They possess an anatomically simple digestive tract, and they are hindgut fermenters and omnivorous. They consume a variety of food items, such as leaves, seeds, insects, roots, fruits, and small vertebrates.

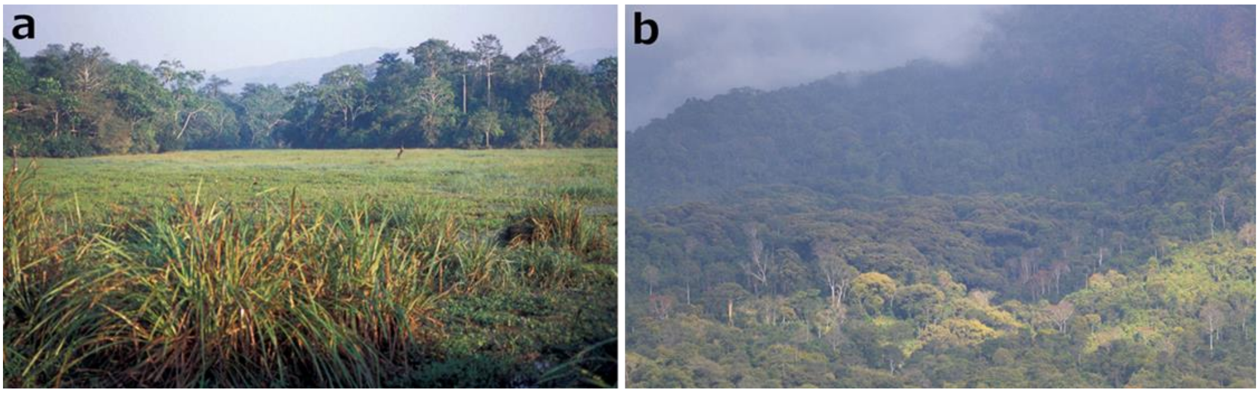

Photo courtesy of Francesco Rovero (**a**) and Rasmus Gren Havmøller (**b**).

Supplementary Figure S2. Views of the two forest blocks considered within the Udzungwa Mountains: Magombera (**a**) and Mwanihana (**b**). The Udzungwa Mountains extend over 19,000 km^2 and represent a mosaic of moist forest blocks of variable sizes (ranging from 12 km^2 to >500 km^2). The forest blocks are interspersed with naturally drier areas and/or habitat modified by

agriculture, human settlements, and logging. The two study forests have contrasting habitat types, altitude, and protection levels: Magombera (**a**) is a small fragment of groundwater and evergreen lowland forest which is separated from other forest blocks by the surrounding intensive agriculture and human settlements. Magombera is not protected and 40% is heavily degraded, which make the forest encroached by nearby villagers for firewood, pole and timber harvesting and occasionally for hunting. Instead, the Mwanihana forest (**b**) is a forest escarpment with forest zones ranging from lowland deciduous to montane evergreen. It is well protected, and it is part of the Udzungwa Mountains National Park (UMNP).

Supplementary Tables

Supplementary Table S1. Generalized Linear Model (GLM) analysis. Regression models testing the relationship between bacterial richness and helminth presence separately for each primate species (Udzungwa red colobus, *Procolobus gordonorum* and yellow baboons, *Papio cynocephalus*) in each forest type, the protected and intact Mwanihana (PF) and unprotected and fragmented Magombera (FF) forests within the Udzungwa Mountains of Tanzania.

Supplementary Table S2. DeSeq2 analysis. Significant differentially abundant bacterial taxa in Udzungwa red colobus (*Procolobus gordonorum*) individuals positive to *Strongyloides* sp. The table reports the SV identifier, the mean of normalized counts (baseMean), the base 2 logarithm of the fold change (log2FoldChange), standard errors of the log2FoldChange (lfcSE), the test statistics (stat), the p-values (pvalue), the corrected p-values (padj) and the predicted taxonomy.

Supplementary Table S3. Generalized Linear Model (GLM) analysis. Regression models testing the relationship between fungal richness and helminth presence separately for each primate species (Udzungwa red colobus, *Procolobus gordonorum* and yellow baboons, *Papio cynocephalus*) in each forest type, the protected and intact Mwanihana (PF) and unprotected and fragmented Magombera (FF) forests within the Udzungwa Mountains of Tanzania.

Supplementary Table S4. DeSeq2 analysis. Significant differentially abundant fungal taxa in Udzungwa red colobus (*Procolobus gordonorum*) individuals positive to *Trichuris* sp. The table reports the SV identifier, the mean of normalized counts (baseMean), the base 2 logarithm of the fold change (log2FoldChange), standard errors of the log2FoldChange (lfcSE), the test statistics (stat), the p-values (pvalue), the corrected p-values (padj) and the predicted taxonomy.
